# Mast Cells and Dendritic Cells as Cellular Immune Checkpoints in Immunotherapy of Solid Tumors

**DOI:** 10.3390/ijms231911080

**Published:** 2022-09-21

**Authors:** Katerina Kalkusova, Sindija Smite, Elea Darras, Pavla Taborska, Dmitry Stakheev, Luca Vannucci, Jirina Bartunkova, Daniel Smrz

**Affiliations:** 1 Department of Immunology, Second Faculty of Medicine, Charles University and Motol University Hospital, V Uvalu 84, 150 06 Prague, Czech Republic; 2 Laboratory of Immunotherapy, Institute of Microbiology of the Czech Academy of Sciences, 142 20 Prague, Czech Republic

**Keywords:** mast cells, dendritic cells, immunotherapy, cellular checkpoint

## Abstract

The immune checkpoint inhibitors have revolutionized cancer immunotherapy. These inhibitors are game changers in many cancers and for many patients, sometimes show unprecedented therapeutic efficacy. However, their therapeutic efficacy is largely limited in many solid tumors where the tumor-controlled immune microenvironment prevents the immune system from efficiently reaching, recognizing, and eliminating cancer cells. The tumor immune microenvironment is largely orchestrated by immune cells through which tumors gain resistance against the immune system. Among these cells are mast cells and dendritic cells. Both cell types possess enormous capabilities to shape the immune microenvironment. These capabilities stage these cells as cellular checkpoints in the immune microenvironment. Regaining control over these cells in the tumor microenvironment can open new avenues for breaking the resistance of solid tumors to immunotherapy. In this review, we will discuss mast cells and dendritic cells in the context of solid tumors and how these immune cells can, alone or in cooperation, modulate the solid tumor resistance to the immune system. We will also discuss how this modulation could be used in novel immunotherapeutic modalities to weaken the solid tumor resistance to the immune system. This weakening could then help other immunotherapeutic modalities engage against these tumors more efficiently.

## 1. Introduction

Cancer is the second most common cause of death globally, and the number of people with the disease increases each year [1,2]. The therapy of cancer currently stands on four pillars––surgery, chemotherapy, radiotherapy, and immunotherapy. The first three pillars (surgery, chemotherapy, radiotherapy) target cancer cells directly. Immunotherapy, on the other hand, is a type of therapy that uses substances to modulate the immune system to help the body fight cancer [3]. For decades, cancer immunotherapy stood aside, not matching the success rate of the traditional treatment modalities (surgery, chemotherapy, or radiotherapy). However, this has changed with immunotherapy drugs called “immune checkpoint inhibitors” [4]. The efficacy of these inhibitors and their successors has already shown unprecedented results for some types of cancer, notably, where the traditional treatment options had already failed [5]. Currently, these inhibitors are increasingly being tested in a large number of clinical trials. For instance, the inhibitors against PD-1 and PD-L1 molecules are tested in thousands of clinical trials in combination with other treatment modalities [6,7,8]. Results of the studies are now allowing these inhibitors to be already registered and used even in first-line cancer therapy [9,10,11], something hardly imaginable a decade ago. Apart from the checkpoint inhibitors, another group of immunotherapeutic modalities showed promising results. These modalities are based on in-vitro-modified/expanded immune cells, which can induce cancer-targeted immune responses [12] or directly target and eliminate cancer cells [13]. Many of these drugs have already been FDA-approved––autologous dendritic cells (DCs) [14] or genetically-modified T cells with chimeric antigen receptors (CAR-T) [15] or T cell receptors [16].

Despite the great success of immunotherapy over the past few years, immunotherapy suffers from cancer resistance. This resistance means that most patients still do not benefit from the therapy [17,18]. Although there are multiple mechanisms of cancer resistance to immunotherapy [19], there could be basically drawn three critical causes why current immunotherapy faces resistance: a limited specificity, an inability to reach and eliminate its targets, and the immunosuppressive microenvironment of solid tumors. The limited specificity is caused by aiming at targets that are not present only on the targeted cancer cells. For instance, the inhibitory checkpoint molecule PD-1 is expressed not only on the tumor-targeting immune cells (such as on cytotoxic CD8^+^ T cells) but also on the tumor-protecting immune cells as regulatory CD4^+^ T cells [20]. Inhibition of PD-1, therefore, simultaneously unleashes both pro- and anti-tumorigenic immune responses, the combination of which may have either a curative or detrimental impact on the state of the disease. This may even happen in the same cohort of the treated patients [20]. The inability of immunotherapy to reach and eliminate its targets is also given by the inability of immune cells to reach and eliminate cancer cells in solid tumors. There are many solid tumors that entirely block their immune cell infiltration and, as such, prevent the immune system from attacking them [21]. The immunosuppressive microenvironment then adds up to the immunotherapy resistance where immunotherapy can overcome the solid tumor barriers, break in, and reach its targets. Herein, the immunosuppressive tumor microenvironment steps in by preventing immunotherapy from further acting against cancer cells efficiently.

To eliminate the resistance requires making solid tumors accessible to the immune cells, overcoming its immunosuppressive microenvironment to allow efficient cancer cell elimination and establishing lasting immune protection from tumor recurrence. These requirements are extremely challenging. However, there are two types of immune cells whose combined harnessing in the immunotherapeutic algorithms could stand up to these challenges. These cell types are mast cells (MCs) and dendritic cells (DCs).

## 2. MCs in Solid Tumors

MCs are tissue-resident highly granulated cells notoriously associated with the pathogenesis of allergic and autoimmune diseases [22,23]. These cells are equipped with a large number of receptors that enable them to respond to a large number of stimuli [24]. These responses include release (exocytosis) of preformed secretory granules (degranulation), migration, or de novo production of biologically and immunologically active products [24]. Their most notoriously known receptor is the high-affinity receptor for IgE, FcεRI. This receptor induces a fast degranulation of MCs, often referred to as anaphylactic degranulation since the content of the released granules are preformed mediators such as histamine, proteases, and heparin [25], which are responsible for anaphylaxis [26,27]. Another receptor that defines MCs is the stem cell factor (SCF) receptor, KIT, which is essential for MC development, survival, and migration [24]. Stimulation through this receptor can also increase or decrease the extent of FcεRI-induced MC degranulation [28,29] or, under some circumstances, even trigger this degranulation [30]. One of the MC receptors that has brought attention in the past several years is the Mas-related G protein-coupled receptor X2 (MRGPRX2) [31]. This receptor can recognize various chemical compounds, and its activation can cause pseudo-allergic reactions [31]. MCs are known to also interact with other immune cells and regulate their functions. Activated MCs can enhance T cell proliferation [32], co-stimulate T cells via a CD28-independent interaction [33], limit viral infection through interactions with γδ T cells [34], attract and affect NK cells [35], and recruit and alter functions of tumor-infiltrating macrophages, myeloid-derived suppressor cells, neutrophils, and dendritic cells [36].

Due to a broad spectrum of MC biological and immunological activities [37], these cells are also considered to be key orchestrators of the tumor immune microenvironment [38]. For that reason, these cells are increasingly scrutinized as potential targets for cancer immunotherapy [39]. However, MCs are highly complex immune cells whose function can render these cells either pro- or anti-tumorigenic. The mechanisms of the pro- or anti-tumorigenic activities of MCs are diverse and involve both the immune and non-immune compartments of the human body [39]. A few examples of these mechanisms shown in Table 1 indicate that effectors of these mechanisms are often soluble cytokines, chemokines, or growth factors. Indeed, the spectrum of these molecules is exceptionally vast and involves all critical molecules used in the regulation of innate and adaptive immunity [40]. However, in addition to the breadth of this spectrum, even one type of the MC-produced effector molecule, such as histamine or the serine protease tryptase (Table 1), was found to show either anti- or pro-tumorigenic activities in dependence on the leading impact they have in individual cancers [41,42,43,44]. The leading anti-tumorigenic impact of individual effector molecules could be the inhibition of tumor growth/tumor cell proliferation [43,45], inhibition of tumor engraftment [46], promotion of DC development and maturation [41], recruitment of CD8^+^ T cells and their subsequent antigen-mediated activation [47], tumor cell cytotoxicity [48], or direct tumor cell clearance [49]. The leading pro-tumorigenic impact, on the other hand, could be the enhancement of tumor cell proliferation [42], angiogenesis [44,50], immunosuppression and inflammation decrease [51,52,53], or promotion of the epithelial-to-mesenchymal transition (EMT) [54].

Such MC functional ambiguity towards tumors makes these cells controversial as to whether these cells should be therapeutically targeted for elimination or, rather, for enhancement of their numbers and activities. Whether MCs become pro- or anti-tumorigenic presumably lies in their functional plasticity, which is already programmed during their development and maturation from stem cell progenitors. Pioneering in vitro studies showed that a sustained presence of different cytokines during MC development programs their activation phenotype [28,55,56]. In dependence on the cytokine presence in the cell culture, this programming can be either reversible [55,56] or irreversible [28]. Since MC progenitors infiltrate tissues in which they then fully develop and mature, thus, acquiring the tissue-specific phenotypes, the tumor microenvironment is presumably also infiltrated with not yet fully developed/matured MCs, and their activation phenotype is then programmed as they develop and mature [57]. The specifics of the tumor microenvironment, such as hypoxia, low pH, and metabolite composition, can then imprint the developing and maturing MCs with a tumor microenvironment-specific activation phenotype, whose impact on the tumor could be hardly inferred from plain numbers of infiltrating MCs and their associations with the disease severity. In addition, mast cells in the tumor microenvironment can be phenotypically/functionally heterogeneous [58]. MCs heterogeneity was initially described based on their localization and protease content. The localization-based division recognizes connective tissue (CTMCs) and mucosal (MMCs) MCs. The protease-content-based division distinguishes MCs subtypes expressing tryptase (MC_T_) or both tryptase and chymase (MC_TC_) [37]. Based on their origin, MCs are also divided into a subgroup of constitutive or inducible MCs [59,60]. Nevertheless, regardless of the yet still enigmatic origin of MCs and their subtype characteristics, the resulting functional heterogeneity of developed and mature MCs seems to be substantially shaped by factors in the surrounding milieu of MC residence [24]. Then different MC functional phenotypes are presumably acquired in inflamed airways [61] and in the microenvironment of different tumors [62]. Indeed, single-cell RNA sequencing studies revealed significant diversity in MC development and MC residence-specific functional characteristics [63]. A recent study showed that MC phenotypic heterogeneity and their histological distribution with potentially distinct functions were observed even within an individual tumor microenvironment [58]. However, since there are currently no known specific markers of the tumor microenvironment-infiltrating MCs and their subtypes, and the MC role towards the tumor can be ambivalent, a design of MC-targeting strategies in cancer immunotherapy is, thus, highly challenging.

## 3. DCs in Solid Tumors

DCs are major professional antigen-presenting cells that present antigens on their surface via major histocompatibility complex I and II (MHC I and MHC II) to T cells to initiate adaptive antigen-dependent immune responses. DCs also mediate cross-talk between the innate and adaptive immune system through direct interactions with other immune cells––natural killer (NK) cells [64], NK T cells [65], neutrophils [66], and MCs [67]. DCs are derived from hematopoietic stem cell progenitors. The progenitors which infiltrate peripheral tissues differentiate into immature DCs [68]. These immature (tissue-resident) DCs have high phagocytic activity of extracellular material [69]. Without a maturation signal, such as stimulation with pathogen-associated molecular pattern (PAMPs) or damage-associated molecular pattern (DAMPs) molecules, the antigen presentation by immature DCs leads to immune tolerance to the presented antigens, including the antigens from the phagocyted material [70]. Such antigen presentation allows the establishment of peripheral immune tolerance to autoantigens [70]. However, once DCs mature, the phagocytic capacity of DC decreases, and DCs start to efficiently present antigens to induce either immune tolerance (tolerogenic DCs) or immune stimulation (immunogenic DCs) [70]. Which phenotype prevails is dependent on the signals triggering the maturation. These signals are elicited by soluble factors or interactions with surrounding cells [71]. Once mature, the phenotype is often irreversible [72].

Human DCs are divided into several subtypes based on their phenotype and ontogeny: plasmacytoid DC (pDC), classical DC (cDC) type 1 and 2 (cDC1 and cDC2), DC3, and monocyte-derived DC (mo-DC) [73]. The pDCs are present in lymphoid organs but not peripheral tissues. Activated pDCs specialize in the production of type I IFNs and are thought to be the main producers of these cytokines during infections and autoimmune diseases [74]. The cDCs are tissue-resident DCs. The cDCs type 1 are specialized in activating CD8^+^ and type 2 on CD4^+^ T cells, both of which play a role in the regulation of anti-tumor immunity [75,76]. The place of their residency then further shapes their phenotype to form multiple subsets of these cells in peripheral tissues [77]. The DC3 subtype is a novel subtype of DCs identified in the blood [78]. DC3s express a monocytic marker CD14, share common markers of cDC2, and have monocyte gene signatures [78,79]. Similar to DC3s are mo-DCs, which show overlapping phenotypes with DC3s [79]. Mo-DCs also share multiple phenotypic markers with monocyte-derived macrophages, thus, making them difficult to distinguish from each other [80]. Mo-DCs are able to in vivo cross-present antigens [81], and their ex vivo production from peripheral blood monocytes has become central to the development of DC-based cancer immunotherapy [82,83].

An important role in the DC anti-tumor activities in the tumor microenvironment is attributed to cDC1s, which play a crucial role in initiating and maintaining anti-tumor immunity by cross-presenting tumor antigens to CD8^+^ T cells, stimulating NK and NKT cells [84]. However, even though their role in anti-tumor immunity is necessary, it seems that it is insufficient and needs to be promoted by a cross-talk with other DC subtypes, namely the cDC2s [85]. The role of cDC2s in tumor immunity is ambivalent as these DCs can show either pro- or anti-tumorigenic activities [76]. Similar is true for mo-DCs and pDCs, both of which can adopt either pro- or anti-tumorigenic activities [86,87]. The mechanism of their pro- or anti-tumorigenic activities is driven by their ability to recruit and stimulate/inhibit cytotoxic or tolerogenic lymphocytes [88]. These abilities are allowed by the production of many chemokines, pro-/anti-inflammatory cytokines, and the expression of immune checkpoint molecules (details reviewed in [87,88]). However, regardless of the DC subtype, the functional phenotype of DCs is significantly shaped by factors in the milieu of their residence [89,90,91,92].

The tumor microenvironment also decides how DCs orchestrate pro- or anti-tumorigenic activities (Table 2). Anti-tumorigenic activities in the tumor microenvironment can be mediated by IL12-producing tumor-infiltrating DCs, which promote CD8^+^ T cell responses [93]; MHC-I cross-dressed DCs presenting tumor antigens and allowing cross-priming to CD8^+^ T cells [94]; CD1d high-expressor DCs, which increase activation of NKT, CD4^+^ and CD8^+^ T cells [95]; or by cCD2s, which can promote control of cytotoxic T cell-resistant tumors via the CD4^+^ T cell-mediated activation of myeloid cells [96]. Mature cDCs and pDCs can also inhibit angiogenesis through the production of IL-12, angiostatic chemokines, or IFNα [97]. On the other hand, pro-tumorigenic activities of DCs can be adopted by PD-1-expressing DCs, which accumulate in the tumor and inactivate CD8^+^ T cells [98]; arginase-expressing DCs, which causes arginine deprivation leading to the inhibition of CD4^+^ T cell proliferation [99] or reactive oxygen species (ROS)-mediated inhibition of CD8^+^ T cells [100]. Pro-tumorigenic activities are also elicited by indoleamine 2,3-dioxygenase(IDO)-expressing DCs, which cause both tryptophan-depletion-mediated inhibition of CD8^+^ T cells and tryptophan-metabolite-mediated expansion of CD4^+^ T_reg_ cells [101]. Other DCs can produce TGF-β to induce immunosuppression [102], make IL10 alongside the surface expression of PD-L1 to impair CD8^+^ T cell activation [103], or shed soluble CD25 to deplete IL-2 and inhibit T cell proliferation [104]. Immature DCs can also promote angiogenesis [105]. Similar to MCs, whether the tumor-microenvironment-elicited DC phenotype drives these cells to pro- or anti-tumorigenic activities could also be hardly inferred from plain numbers of the infiltrating DCs and their associations with the disease severity. Even though recent technological advances allow for detailed characterization of DC subtypes infiltrating tumors [106,107], there are no known markers that would enable specific targeting of individual DC subsets in the tumor microenvironment to enhance or suppress, respectively, their pro- or anti-tumorigenic activities.

## 4. MC/DC Interplay

Both MCs and DCs are powerful standalone players in the tumor microenvironment. However, both cell types were also shown to impact each other. This impact can occur either via secreted factors or direct interactions. The interaction via secreted factors is less specific and driven mainly by MCs towards DCs. MC secreted factors can promote tolerogenic [52] or immunostimulatory [108] functions of DCs. On the other hand, direct interactions between these cell types are more specific and can impact both cell types. A study showed that migratory DCs in inflamed skin initially scan MCs and later come into long-lasting synaptic interactions. These interactions result in a DC-to-MC molecule transfer, including major histocompatibility complex class II (MHCII) molecules, which then enables MCs to prime T cells, therefore, promoting T-cell-driven inflammation in the skin [109]. Vice versa, activated MCs can form immunological synapses with immature DCs and transfer the MC-internalized antigen to immature DCs. The transferred antigen is then processed and presented by DCs to stimulate T cells [67]. Additionally, the tolerogenic or stimulatory outcome of the MC–DC interaction can be substantially affected by the expression of immune checkpoint molecules and their ligands on both cell types [53].

Regardless of the mechanisms that come into play upon MC–DC interactions, these mechanisms can substantially impact the balance between the tolerogenic and stimulatory activities of the immune system. Indeed, MCs activity toward DCs was shown to substantially suppress [52,110] or, vice versa, promote the immunogenic properties of DCs [111,112]. The suppression was elicited by MC-produced IL-13, which inhibited IL-12 expression in DCs with subsequent inhibition of DC-mediated induction of the T_H_1 response [52], or by promoting the DC tolerogenic phenotype through MC-produced large amounts of GM-CSF [110]. On the other hand, the promotion was elicited either by DC engulfment of MC-released granules, which was followed by DC migration to lymph nodes, their maturation, and boosting their T-cell priming efficiency [108], or by MC-mediated DC maturation followed by their recruitment to the site of infection [112].

## 5. MCs in Immunotherapy of Solid Tumors

The ambivalent role of MCs in tumors makes these cells challenging to judge how these cells should be targeted. Recent studies show that depletion of these cells or downregulation of their functions in the tumor microenvironment can help break tumor resistance [113,114,115]. Although these studies were made in mouse models, they used MC-targeting drugs that are already used in clinical practice––antihistamine and MC stabilizer ketotifen [116,117], MC stabilizer cromolyn [117], or c-KIT inhibitors [118]. Their use, or use of other clinically administered or tested MC-targeting drugs (anti-Siglec therapies [119] or other biologics [115]), could in combination with other cancer treatment modalities (i.e., chemotherapy or immunotherapy) then indeed represent a new promising treatment strategy in which MCs would be approached as inhibitory immune checkpoint cells in the tumor microenvironment [113,114]. This therapeutic approach would be presumably relevant in tumors where the tumor burden with MCs or their products are largely and convincingly associated with the disease severity and dismal prognosis [120,121], or where another therapeutic modality increases their numbers with pro-tumorigenic activities and causes a resistance to the therapy [122].

On the other hand, many studies showed that tumoral/peritumoral MCs are not only associated with a better prognosis [123,124] but that MCs can also directly eliminate cancer cells [49,125]. The mechanism of this elimination could be even unique, as demonstrated in a recent study where MCs were found to efficiently kill cancer cells with the help of cancer-cell-penetrating tunneling nanotube-like structures [48]. This killing was preceded by the recognition of cancer cells through the cancer cell antigen-specific IgE bound to the FcεRI expressed on the surface of MCs [48]. In addition, another recent study of the group showed that the IgE-sensitized MCs could easily infiltrate and eliminate masses of cancer cells both in vitro and in vivo [126,127]. This infiltration is presumably driven by MC chemotaxis towards the antigen of the FcεRI-bound IgE [128]. This antigen-driven chemotaxis and the unique killing mechanism may provide these cells with a novel advantage over other immune cells, which can also recognize and kill cancer cells [129]. Indeed, with the technological advances in ex vivo preparation of MCs from adipose tissue or CD34^+^ hematopoietic progenitors, a new field is already opening where MCs in combination with tumor antigen-specific IgEs are used for adoptive cell transfer (ACT)-based cancer cellular immunotherapy [130]. MCs in this field then could newly join the list of immune cells used or tested for this therapeutic modality.

## 6. DCs in Immunotherapy of Solid Tumors

In contrast to MCs, many cancer immunotherapeutic strategies based on DCs have already been tested in the last few decades. These strategies are mainly based on the unique ability of DCs to shape both adaptive and innate immune responses. As professional antigen-presenting cells, DCs are able to induce tumor-antigen-specific responses through the interaction with naive T cells [131]. Furthermore, mature DCs produce cytokines such as interleukin-12 or type I interferons that enable the regulation of many immune cell types that take part in anti-tumor immunity, including many innate immune cells [132,133]. For these biological features, DCs were believed to be very promising tools for ACT-based cancer cellular immunotherapy, also called––DC-based vaccines. DC-based vaccines are usually based on autologous DCs that are differentiated ex vivo from monocytes or hematopoietic stem cell progenitors, loaded with tumor antigens, and exposed to maturation stimuli. DCs prepared in this way are then transferred into the patient’s body, where they induce anti-tumor immune responses through the activation of tumor-specific lymphocytes [134].

The first DC-based cancer vaccine clinical trials date back to the 1990s of the twentieth century. These early clinical trials mainly focused on the treatment of melanoma and showed promising results and favorable safety profiles [135,136,137]. The next wave of trials focused on leukemia and other solid tumors [138,139,140]. Out of so many clinical trials, there came only one FDA-approved DC-like-based commercially available product, Sipuleucel T (Provenge), which was designated for the treatment of metastatic prostate cancer [141]. However, this product is still not widely used in clinical practice [142].

The disappointment from the early “DC era” is presumably due to the fact that many studies were not designed in combination with other immunotherapeutic interventions and patients were enrolled at late stages of the disease, often associated with advanced tumor microenvironment and the disease-elicited immune system defects [143,144,145]. However, DC-based immunotherapy still goes on through efforts to find new ways for their improvement [146]. Due to advances in genomics, many recent DC-based approaches focus on targeting neoantigens that have become considered powerful targets for cancer immunotherapy [147,148,149], including DC-based vaccines [150]. In addition, current designs of therapeutic algorithms also rely on combinations of DC-based cancer vaccines with other therapeutic modalities [151,152]. There is also a developing new field that attempts to identify new biomarkers that could predict the therapeutic efficacy of the vaccines based on the patient’s immunotype [153].

## 7. MC/DC Interplay as the Cellular Immune Checkpoint for Cancer Immunotherapy

The history of the use of MCs and DCs in cancer immunotherapy is, therefore, entirely different. Whereas MCs have become the center of interest in immunotherapy recently [130], DCs are not new players in the field. Three decades ago, DCs were thought to be highly promising immune cells for cancer therapy, namely, for ACT-based immunotherapy. Despite many disappointments in clinical trials, the use of these cells still showed a notable therapeutic efficacy, and their role in cancer immunotherapy is indeed still ongoing [146,154,155]. As research into immunotherapy accelerated after the immune checkpoint inhibitor had entered the field, the efficacy of DC-based immunotherapy was found to be largely restricted by the settings (immunotype) of the patient’s tumor immune microenvironment [153,156]. Moreover, there is growing evidence that the functional heterogeneity of DC populations is even more complex than previously thought [73], and, therefore, more attention needs to be paid to the way DCs are ex vivo produced for ACT [82]. This attention relates to the DC differentiation process, antigen loading strategy, and DC maturation [82,146]. In this regard, the interaction of MCs with DCs may come with an advantage in the ex vivo preparation of DCs. In our previous work, we found that a stimulated human LAD2 MC line could be a highly potent cellular adjuvant for the maturation of ex-vivo-produced human-monocyte-derived DCs for ACT [157]. This high adjuvant potency was conditioned by previous MC stimulation with thapsigargin, an inhibitor of sarco/endoplasmic reticulum Ca^2+^-ATPases (SERCA) [158,159], which induces a strong mobilization of intracellular calcium followed by fast and extensive degranulation of LAD2 MCs [160]. Such previously stimulated and rinsed LAD2 MCs were, unlike their non-stimulated or FcεRI-stimulated counterparts, able to mature monocyte-derived DCs, which were even refractory to the maturation with polyinosinic:polycytidylic acid (poly I:C), a toll-like receptor-3 agonist often used for the production of matured DCs for ACT-based cancer cellular immunotherapy [154,155]. Moreover, the study showed that the adjuvant effect was still notable even when the MC:DC ratio was 1:150 [157]. This maturation efficiency indicated that a very small number of MCs can promote DC maturation. Apart from maturation, the MC–DC interaction can provide a targeted loading of DCs with an extracellular antigen, which is then efficiently presented to T cells [67]. This mechanism could be presumed to come also into play upon an MC-based ACT, where MCs could be expected not to only specifically (in an antigen-dependent manner) destroy cancer cells in the tumor [126] but to also come in contact with the tumor-infiltrating DCs and restore their antitumor activity [67]. Restoration of efficient antitumor activity of otherwise defective tumor-infiltrating DCs was already demonstrated for many tumors using different approaches [98,161,162]. Whether an MC-based ACT could also participate in the DC restoration in the tumor microenvironment and, as such, promote lasting immune protection against the disease remains to be investigated.

## 8. Future Perspectives

**Figure 1:**
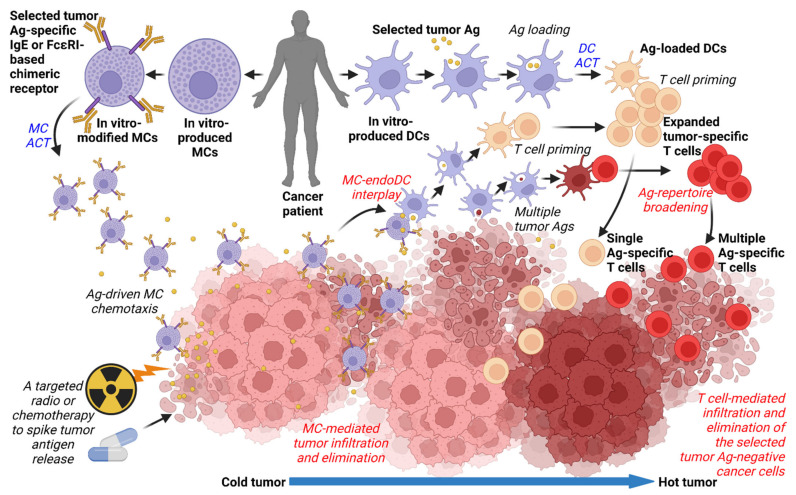
The perspective of combined MC- and DC-based ACT. Autologous MCs are produced in vitro from adipose tissue or CD34^+^-derived stem cell progenitors. DCs are produced in vitro from peripheral blood monocytes or CD34^+^-derived stem cell progenitors. MCs are sensitized with a selected tumor antigen (Ag)-specific IgE or genetically modified with a chimeric FcεRI-based receptor specific to the selected tumor Ag. The IgE-sensitized or genetically modified MCs are transferred back to the patients (MC ACT). The transferred MCs are specifically chemoattracted to the tumor through the selected Ag concentration gradient. The release of the selected Ag from the tumor is spiked by targeted radiotherapy or chemotherapy. Chemoattracted MCs infiltrate the tumor mass where they eliminate cancer cells. The tumor-infiltrating MCs also interact with endogenous(endo)/tumor-infiltrating DCs to load them with the tumor Ag and restore their antitumor activity through priming tumor Ag-specific T cells. Multiple tumor Ags, including the patient-specific neoAgs, are released from the tumor and captured by endogenous DCs, which primes T cells with a broad repertoire of tumor Ag specificity. The DC-primed T cells expand and infiltrate the tumor. The infiltrated multiple Ag-specific T cells also eliminate evasive variants of cancer cells that are negative for the selected tumor Ag. To corroborate the efficacy of the MC-based ACT, autologous DCs are produced in vitro from peripheral blood monocytes or CD34^+^-derived stem cell progenitors. The DCs are loaded with the selected tumor antigen or other tumor-associated Ags, matured, and transferred back to the patients (DC ACT). The transferred DCs promote the expansion of the tumor-Ag-specific T cells. The previously cold tumor turns into a hot tumor by infiltrating immune cells with prevailing antitumor activities. Created with BioRender.com (agreement number: HM24B8W5CT).

Recent development in MC research and its translation to cancer immunotherapy opens new avenues in developing novel immunotherapeutic combination strategies for treating solid tumors. The opening of the avenues was enabled by novel approaches in ex vivo clinical-scale preparation of autologous MCs [130,163,164]. The opening was then secured by the findings that these cells can not only kill tumor cells in an antigen/IgE/FcεRI-dependent manner but also translocate in vivo into tumors where they can deliver a therapeutic impact [126]. This translocation into tumors, presumably based on antigen-driven chemotaxis of MCs, seems to be one of the MC attributes that could challenge solid tumor resistance to ACT-based cancer immunotherapy. In addition, it could be even enhanced after targeted radiotherapy or chemotherapy, which spikes the release of tumor antigens from dying cancer cells [165,166] (Figure 1).

Apart from the engagement of IgE-mediated mechanisms in the MC antitumor activities, MCs can also enter a new uncharted territory of advanced genetic engineering that has already been developed for years for ex vivo modifications of other immune cells [167]. Similar to the T cell chimeric receptors, CAR [168] and TCR [169], chimeric antigen receptors based on FcεRI and its downstream signaling could be one of the plausible strategies for advanced MC-based ACT (Figure 1).

As a standalone produced/engineered player for ACT, MCs would certainly suffer from the drawback apparent to other immune cells used for ACT––the single antigen-driven specificity, which could be evaded by the loss or downregulation of the expression of the targeted tumor antigen [170]. This drawback could be, however, overcome through the MC–DC interplay. This interplay can harness endogenous or even tumor-infiltrating DCs whose engagement and loading with antigens released from MC-killed cancer cells could stimulate T cells and, as such, broaden the tumor antigen repertoire through which the immune system recognizes and eliminates cancer cells [134,171,172]. This repertoire could also include neoantigens, whose targeting by T cells seems to be critical for immunotherapeutic efficacy [147]. This presumed synergistic MC–DC-interplay-based antitumor activity would be particularly important in so called “cold tumors” [21], which prevent the immune cells from the tumor infiltration and subsequent induction of broad antigen-driven antitumor immune responses. Since the initial work with the cytotoxic MCs was performed with the Her2^+^ breast cell line [126], which forms “cold tumors” [173], there is a likelihood that the presumed MC–DC-interplay-elicited antitumor activity could also be attained in the immunotherapeutically-difficult-to-challenge “cold tumors” [21,174] (Figure 1).

Many combination strategies in cancer therapy rely on DC ACT as enhancers of therapeutic efficacy [146]. Many of these strategies involve combinations of DC ACT with other immunotherapeutic approaches, including immune checkpoint inhibitors [174], NK cells [175,176], or CAR-T cells [177]. In cold tumors, the combination is presumed to work towards turning the “cold tumors” into “hot tumors,” thus, enhancing the tumor immune cell infiltration [178]. Combining the cold-tumor-penetrating cytotoxic MCs with DC ACT could, therefore, also synergize in promoting the cold-to-hot turning process (Figure 1). Apart from the “turning process”, both cell types could also enhance the anti-tumor activity of other immune cells, including endogenous T cells [32] or adoptively transferred CAR-T cells [177].

Using MCs in combination with DCs in cancer immunotherapy needs to be designed with respect to tumor types, ongoing therapy, and mast cell-driven co-morbidities. The tumors that are known to be more sinister when large numbers of MCs infiltrate them [179] or when immunotherapy enhances their numbers alongside the growing tumor resistance [122] are presumably situations where MCs activities need to be suppressed or the cells even eliminated. A similar approach needs to be also considered when patients suffer from diseases related to dysregulated MC numbers [180] or their activation [181] or when MCs are directly part of the malignant neoplastic disease [182].

## 9. Concluding Remarks

MCs are now becoming a new player in the field of cancer immunotherapy. The new advances in their in vitro preparation and the recent demonstration of MC’s potential to infiltrate solid tumors and kill cancer cells within the tumor [126] bring expectations in these cells for cancer immunotherapy [130]. Although the demonstrated performance of the cancer cell-killing MCs in the tumor needs to be further investigated, this review suggests that the therapeutic power of MCs in cancer treatment may not so much lie in their single-antigen-driven cytotoxicity, but in their potential to trigger much more robust and lasting anti-tumor immunity to those solid tumors that so far have resisted immunotherapeutic interventions. This review also suggests that the key partner necessary for unleashing the therapeutic potential of MCs are DCs and that combination of MC- and DC-based ACT could be the therapeutic algorithm for licensing MCs for efficient cancer immunotherapy in clinics.

## Figures and Tables

**Table 1:** The impact of MCs on the tumor microenvironment.

Effector	Impact	Malignancy/Model	Reference
**Anti-tumorigenic MCs**			
Tryptase	Inhibition of tumor cell proliferation	Melanoma	[43]
Histamine	Promotion of DC development and maturation	Lymphoma	[41]
IL-6	Inhibition of tumor growth	Melanoma	[45]
Nanotubes/TNF-α	Cytotoxic activity	Breast cancer	[48]
IL-9-mediated MC activity	Tumor engraftment inhibition	Colon carcinoma	[46]
CCR2, CCR7, Leukotriene B4	CD8^+^ T cell recruitment and antigen-mediated activation	Intestinal tumors	[47]
Phagocytosis	Tumor cell clearance	Breast cancer	[49]
**Pro-tumorigenic MCs**			
Tryptase	Promotion of angiogenesis	Pancreatic cancer	[44]
Histamine	Enhanced proliferation of histamine receptor R1^+^ tumor cells	Hepatocellular carcinoma	[42]
VEGF	Promotion of angiogenesis	Laryngeal squamous cell carcinoma	[50]
IL-10	Anti-inflammatory/immunosuppressive	-	[51]
IL-13	Anti-inflammatory/immunosuppressive	-	[52]
IL-8	Promotion of EMT	Thyroid cancer	[54]
PD-1	Induction of IDO^+^ tolerogenic DCs	-	[53]

**Table 2:** The impact of DCs on the tumor microenvironment.

Effector/Marker	Impact	Reference
**Anti-tumorigenic DCs**		
IL-12^+^ DCs	Promotes CD8^+^ T cell responses	[93]
DC cross-dressing	CD8^+^ T cell priming	[94]
CD1d^++^ DCs	Increased activation of NK T cells, CD4^+^, and CD8^+^ T cells	[95]
cDC2	Increased control of cytotoxic T-cell-resistant tumors via CD4^+^ T-cell-mediated activation of myeloid cells	[96]
Mature cDCs and pDC	Angiogenesis inhibition	[97]
**Pro-tumorigenic DCs**		
PD-1^+^ DCs	CD8^+^ T cell inactivation	[98]
Arginase^+^ DCs	Arginine-deprivation-mediated inhibition of CD4^+^ T cell proliferation ROS-mediated inhibition of CD8^+^ T cells	[99] [100]
IDO^+^ DCs	Tryptophan-depletion-mediated inhibition of CD8^+^ T cells Tryptophan-metabolite-mediated expansion of CD4^+^ T_reg_ cells	[101]
TGF-β^+^ DCs	Anti-inflammatory/immunosuppressive	[102]
IL-10^+^/PD-L1^+^ DCs	Impaired CD8^+^ T cell activation	[103]
sIL25^+^ DCs	Inhibition of T cell proliferation by IL-2 depletion	[104]
Immature DCs	Angiogenesis promotion	[105]

## Data Availability

Not applicable.
